# Association of geriatric assessment with multidisciplinary treatment recommendations and treatment concordance among older patients with breast cancer

**DOI:** 10.3389/fonc.2026.1892469

**Published:** 2026-10-20

**Authors:** Yihua Jin, Jing Yu, Xu Zhang, Siji Zhu, Kunwei Shen, Xiaosong Chen

**Affiliations:** Department of General Surgery, Comprehensive Breast Health Center, Ruijin Hospital, Shanghai Jiao Tong University School of Medicine, Shanghai, China

**Keywords:** adjuvant therapy, breast cancer, geriatric assessment, multidisciplinary team, older patients, treatment concordance

## Abstract

**Background:**

Multidisciplinary team (MDT) strategies and geriatric assessment (GA) are widely used in cancer treatment to guide more individualized treatment recommendations and improve prognosis.

**Methods:**

In this retrospective analysis of a prospectively collected observational cohort, we evaluated the association of GA parameters with MDT treatment recommendations and with patient concordance with those recommendations in older patients with breast cancer. A total of 211 patients aged ≥60 years were enrolled. GA was assessed using nine instruments before surgery, and adjuvant treatment decisions were made by MDT discussions. Associations between GA parameters and MDT treatment recommendations, and concordance with those recommendations, were analyzed.

**Results:**

Patients with lower Vulnerable Elders Survey (VES-13) scores (better status) (95.7% vs. 77.8%, *p* = 0.021) and higher Barthel Index (BI) scores (better status) (96.7% vs. 75%, *p* = 0.003) were more frequently recommended adjuvant radiotherapy. The rate of adjuvant chemotherapy recommendation was also higher for patients with lower VES-13 scores (46.8% vs. 21.1%, *p* = 0.004) and higher BI scores (45.6% vs. 27.5%, *p* = 0.037). Concordance with the recommended adjuvant chemotherapy was higher among patients with lower modified Charlson Comorbidity Index (CCI) scores (better status; CCI without age points) (80.3% vs. 53.8%, *p* = 0.038).

**Conclusion:**

These findings indicate that GA parameters were associated with MDT treatment recommendations and with concordance with recommended adjuvant chemotherapy in older patients with breast cancer.

## 1 Introduction

Breast cancer is the most frequently diagnosed malignancy among women worldwide. In 2026, an estimated 321,910 new cases and 61,950 deaths are projected in the United States (1). The mortality rate of breast cancer continues to increase with age (2). Considering the complexity of cancer care, multidisciplinary team (MDT) strategies are widely used to improve coordination, communication, and decision-making between health care team members and patients (3). Adding an MDT discussion before chemotherapy initiation may reduce the incidence of high-grade chemotherapy-related toxicity in older patients with breast cancer (4). Patients with locally advanced breast cancer also benefit from MDT management, with better local control and fewer relapses (5). A study conducted at our center reported that concordance with MDT recommendations was closely related to overall survival (OS) and disease-free survival (DFS) (6). However, older patients with breast cancer tended to show poor concordance (6, 7).

Older patients are a highly heterogeneous group, characterized by many age-related conditions (8). On the one hand, senescence remodels the tumor microenvironment, thus influencing the therapy response (9). On the other hand, cognitive impairment, reduced mobility, and multimorbidity become more common with aging (10). Comorbidity has been closely associated with lower concordance with treatment recommendations (11). Moreover, an increasing body of research focuses on undertreatment and overtreatment due to heterogeneity of older patients with cancer (12). As life expectancy increases, the gap has widened between the growing number of older patients with breast cancer and the persistent scarcity of clinical trial data in this population (13). Geriatric assessment (GA), as a pivotal tool to uncover vulnerabilities and needs of older patients, may help to guide personalized interventions and improve prognosis (14).

The importance of integrating GA into oncology clinical care has been reiterated by the American Society of Clinical Oncology (ASCO), the International Society of Geriatric Oncology (SIOG), and the European Society of Breast Cancer Specialists (EUSOMA) (8, 15). As proposed by Chapman et al., five distinct delivery models have been summarized to implement GA within oncology clinical workflows: the screen-and-refer model, shared care model, multidisciplinary consultative model, geriatrician-embedded consultative model, and self-administered GA model (16). The first four delivery models rely on in-person evaluation delivered by specialized geriatric providers. By contrast, the self-administered GA model adopts patient-completed questionnaires as the sole mode of evaluation, without mandatory on-site geriatrician participation (17, 18). Digital tools such as smartphone mini-programs can be integrated into this model to streamline screening procedures (19). In the present study, GA was based on patient-completed questionnaires administered with the on-site guidance of specialized breast nurses, rather than a geriatrician-led comprehensive evaluation.

Across global oncology settings, GA has proved valuable for mortality prediction, treatment plan individualization, patient-reported outcomes, and possible cost-effectiveness (20). However, only a small proportion of oncologists use GA in routine clinical practice, probably owing to lack of knowledge or training (21). Therefore, there is still an urgent need for more research to demonstrate the necessity of GA.

The primary objective of this study was to evaluate the GA status of older patients with breast cancer and to explore the associations between multi-domain GA parameters and MDT recommendations for adjuvant treatment and patient concordance with those recommendations.

## 2 Materials and methods

### 2.1 Participants and clinical data acquisition

This study was a retrospective analysis of prospectively collected data. The present analysis was approved by the Ethics Committee of Ruijin Hospital, SJTU School of Medicine (approval number 2020-309; 15 July 2020). All participants signed written informed consent following the Declaration of Helsinki. Participants were recruited via consecutive non-random sampling during the study period. Patients were prospectively recruited between June 2019 and January 2020 from Shanghai Ruijin Hospital. An age of 60 years was adopted as the inclusion threshold, consistent with the definition of older adults commonly used in China and in geriatric oncology studies (22, 23). The eligibility criteria were as follows: patients aged ≥60 years; curative surgery for breast cancer after recruitment; and attendance at the postoperative MDT discussion. Patients were excluded from the study if they had advanced or recurrent disease, refused biopsy for pathological diagnosis, were diagnosed with benign lesion, were unable to tolerate or declined surgery, or had undergone curative surgery before GA survey. Clinicopathological characteristics including medical history of cancer, family history of breast cancer, pathologic type, histologic grade, tumor size, lymph node involvement, and expression of estrogen receptor (ER), progesterone receptor (PR), human epidermal growth factor receptor 2 (HER2), and Ki-67 were extracted from the Shanghai Jiao Tong University Breast Cancer Database. The cutoff point for ER and PR expression was set at 1%. Meanwhile, tumors were considered as HER2-positive only if scored 3+ by immunohistochemistry (IHC) or 2+ by IHC and amplified by fluorescence *in situ* hybridization (FISH). In addition, treatment details including type of surgery, MDT advice, adjuvant treatment received, and the follow-up information were also retrieved.

### 2.2 Geriatric assessment

Each participant was asked to complete a panel of GA tools through a smartphone app, which included the following: Vulnerable Elders Survey (VES-13) (24), Barthel Index (BI) (25), Eastern Cooperative Oncology Group Performance Status (ECOG PS) (26), Activities of Daily Living (ADL) (27), Instrumental Activities of Daily Living (IADL) (27), 6-Item Cognitive Impairment Test (6-CIT) (28), Charlson’s Comorbidity Index (CCI) (29), American Society of Anesthesiologists Score (ASA Score) (30), and Geriatric-8 (G8) (31). Because age is itself a component of the CCI, the age-related points were subtracted to obtain a modified CCI score (CCI without age points), so that comorbidity could be assessed independently of age.

Cutoff values for VES-13 and ECOG PS were adopted from a previously published study (32). For G8 and 6-CIT, stratification followed each questionnaire’s original suggested criteria. For G8, the original scale-defined threshold was applied. For BI, ADL, IADL, the modified CCI score, and ASA score, patients were dichotomized based on the median score derived from our present study cohort. Cutoffs adopted from the original instruments or from published studies are referred to as *a priori* cutoffs, whereas those derived from the cohort distribution are study-derived cutoffs. Of note, higher scores indicate worse status for VES-13, ECOG PS, 6-CIT, the modified CCI score, and ASA score, whereas higher scores reflect better status for BI, ADL, IADL, and G8. Detailed scale score ranges and cutoff source for each scale are provided in **Supplementary Table S1**. Full item lists of the GA questionnaires are provided in **Supplementary Appendix S1**.

GA was performed on-site in hospital before curative surgery. Patients were notified by specialized breast nurses at admission to complete questionnaires via an in-house smartphone mini-program, which is not a commercial product and serves routine clinical workflows including scale collection and treatment reminders. Specialized nurses provided one-on-one operational guidance during assessment. Patients answered items independently whenever possible; for participants with cognitive impairment, nurses assisted with item interpretation and answer recording, while family members were not routinely involved. Scores for ECOG PS and ASA were assigned by the attending clinicians, and validated Chinese versions of all instruments were used. The smartphone mini-program used for GA is the same system described in our previous report (7) and is integrated into routine care, including treatment reminders and nurse support.

### 2.3 MDT discussion

At our center, patients with early breast cancer who undergo surgery are routinely reviewed by an MDT panel to define an individualized adjuvant treatment plan; the composition of the panel, the procedure of the MDT meeting, and the treatments considered have been described previously (6). In the present study, results from the GA were not shared with MDT panel members. GA data were collected prospectively within the routine clinical workflow of our center and ran in parallel with the MDT workflow; the data were analyzed retrospectively for the present study and did not inform or modify real-time MDT treatment recommendations. No geriatrician was formally part of the MDT panel. For patients with complex comorbidities, discipline-specific specialists could be consulted according to routine clinical practice, though this consultation was not standardized within the study protocol. A complete treatment recommendation is offered to patients after the MDT discussion, including radiotherapy (yes or no), chemotherapy (yes or no, drugs and cycles), endocrine therapy [yes or no, selective estrogen receptor modulator (SERM) or aromatase inhibitor (AI), with or without ovarian function suppression (OFS)], and anti-HER2 therapy (yes or no, trastuzumab alone or with pertuzumab).

Radiotherapy was only discussed among patients with T3 tumors, positive lymph node metastasis, or patients receiving breast-conserving surgery. Chemotherapy was discussed among all patients. Endocrine therapy was only discussed among patients with hormone receptor (HR)-positive tumors, and anti-HER2 therapy was discussed among patients with HER2-positive breast cancer.

### 2.4 Measurement of concordance with the MDT recommendation

Concordance with the MDT recommendation was defined as agreement between the treatment actually received and the treatment recommended by the MDT. For each modality (radiotherapy, chemotherapy, endocrine therapy, and anti-HER2 therapy), concordance was assessed only among patients for whom that treatment was recommended. Any discrepancy between the treatment received and the recommendation—including not starting the recommended treatment, receiving a different regimen or dose, or not completing the planned course—was recorded as non-concordance. Initiation, treatment modification, and early discontinuation were not recorded as separate endpoints, and persistence with the planned 5-year course of endocrine therapy was not evaluated. This endpoint describes agreement between the MDT decision and actual clinical management and is not intended as a judgment of patient behavior. The reasons for a discrepancy, such as toxicity or patient preference, were not systematically collected. Overall concordance was defined as concordance with all recommended modalities. Concordance was assessed from outpatient records and structured telephone interviews performed by specialist nurses during postoperative follow-up.

### 2.5 Statistical considerations

No formal blinding was performed for this observational cohort; all analyses used de-identified datasets for privacy protection. Clinicopathological data were retrieved from the institutional Shanghai Jiao Tong University Breast Cancer Database, and GA data from our in-house smartphone mini-program were merged using de-identified study IDs. Patients with missing individual geriatric scale data remained in the overall cohort and were excluded only from analyses pertaining to that scale. Only those with critical missing core clinicopathological endpoints were excluded from the final cohort.

Clinicopathological characteristics were presented as patient number and percentage [*n*, (%)]. Results of GA were presented as categorical variables. Distributions of the GA among different age groups as well as patients’ concordance according to GA results were compared using Fisher’s exact test. Two-sided *p*-values <0.05 were considered statistically significant. Analysis was conducted using SPSS version 26 (SPSS Inc., Chicago, IL, USA) for Mac and figures were created using GraphPad Prism 9 (GraphPad Prism, Inc., San Diego, CA, USA).

The primary objective of this study was to explore the associations between multi-domain GA parameters and MDT recommendations for adjuvant treatment and patient concordance with those recommendations. Because this was an exploratory, hypothesis-generating study, no single primary endpoint was pre-specified; the main outcomes were the MDT recommendation for each adjuvant modality and concordance with each recommendation. The remaining comparisons are exploratory, and no formal correction for multiple testing was applied.

## 3 Results

### 3.1 Basic characteristics

Overall, 232 patients with breast cancer were assessed for eligibility; 216 underwent curative surgery and 211 attended the postoperative MDT and were included in the analysis (**Figure 1**). Among these 211 patients, 207 were women and 4 were men, and the median age was 71 years [interquartile range (IQR) 67–76; range 63–90]. A total of 25 patients had a breast-related medical history, including 3 with a history of breast cancer. The numbers of patients aged 60–69, 70–79, and ≥80 years were 87 (41.2%), 102 (48.3%), and 22 (10.4%), respectively. Clinicopathological characteristics were generally comparable across the three age groups and are summarized in **Supplementary Table S2**.

**Figure 1:**
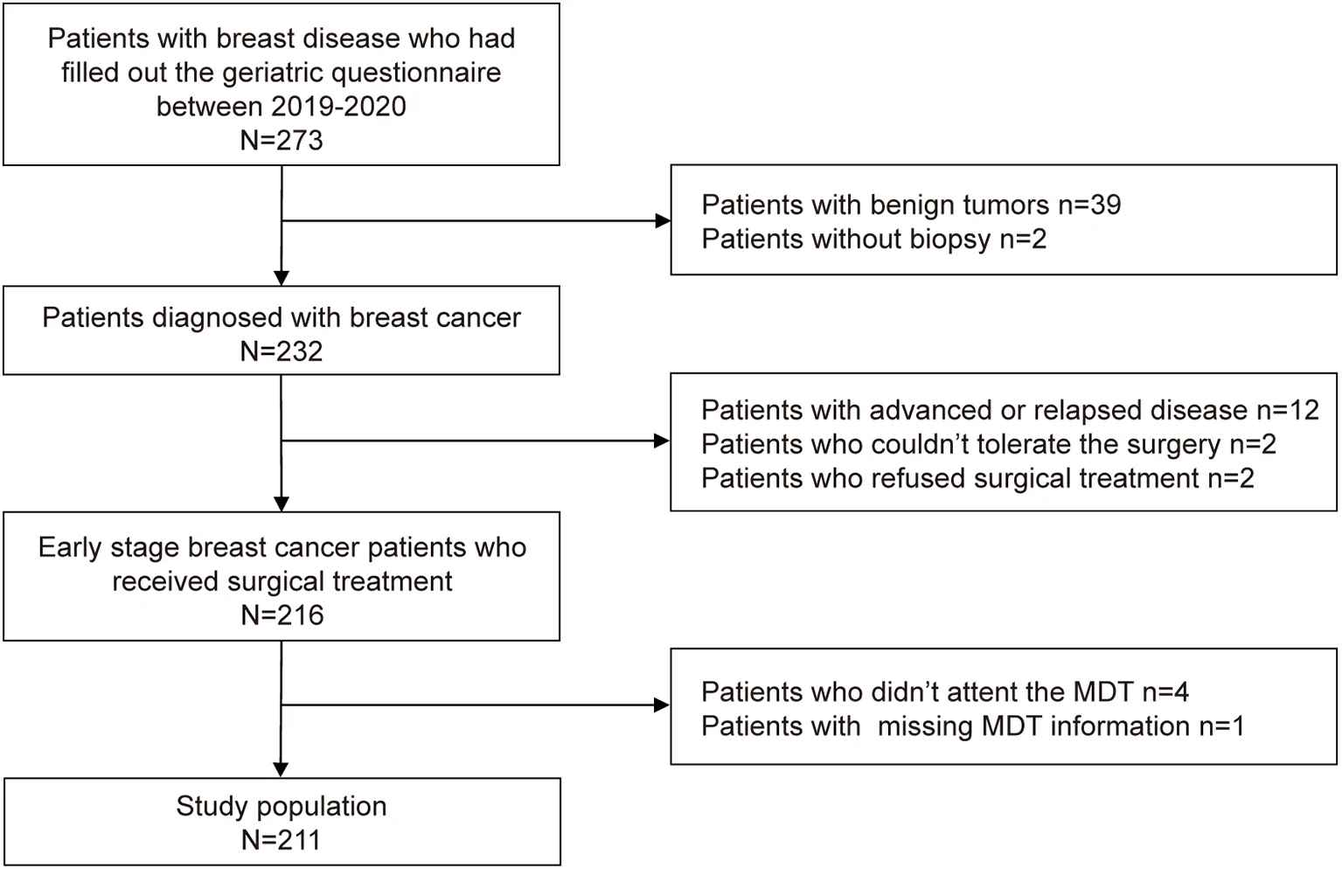
Flowchart of enrollment.

### 3.2 GA parameter scores according to age

Scores of certain GAs were significantly different among age groups, including VES-13, BI, ADL, IADL, and 6-CIT. Regarding VES-13, the proportion of patients with worse status (higher scores) increased with age (5.7%, 18.6%, and 63.6% in the 60–69-year, 70–79-year, and ≥80-year age groups, respectively; *p* < 0.001; **Supplementary Table S3**). For the BI, worse status (lower scores) became more frequent with increasing age (10.3%, 17.6%, and 59.1%; *p* < 0.001; **Supplementary Table S3**), and a similar pattern was observed for ADL (18.4%, 28.4%, and 68.2%; *p* < 0.001; **Supplementary Table S3**) and IADL (32.2%, 48.0%, and 68.2%; *p* = 0.004; **Supplementary Table S3**). For 6-CIT, the proportion of patients with worse status (higher scores) also increased with age (11.5%, 17.6%, and 31.8%; *p* = 0.045; **Supplementary Table S3**).

### 3.3 Surgery choices according to age and GA

Among the 211 eligible patients, 150 (71.1%) underwent mastectomy and 61 (28.9%) underwent breast-conserving surgery. The proportion of patients undergoing breast-conserving surgery did not differ significantly across age groups: 26/87 (29.9%), 26/102 (25.5%), and 9/22 (40.9%) in patients aged 60–69, 70–79, and ≥80 years, respectively (*p* = 0.339). Axillary surgery was performed in 199/211 patients (94.3%), comprising sentinel lymph node biopsy (SLNB) in 149/211 patients (70.6%) and axillary lymph node dissection (ALND) in 137/211 patients (64.9%). Among them, 87 patients underwent both procedures, 62 patients underwent SLNB alone and 50 patients underwent ALND alone. SLNB was performed in 63/87 (72.4%), 73/102 (71.6%), and 13/22 (59.1%) patients aged 60–69, 70–79, and ≥80 years, respectively (*p* = 0.452), and ALND was performed in 55/87 (63.2%), 70/102 (68.6%), and 12/22 (54.5%), respectively (*p* = 0.414).

There was no statistically significant difference in the choice of breast surgery among different groups of GA scores. However, the choice of lymph node surgery differed according to the scores of ADL, IADL, and ASA score. Patients with better functional status (higher ADL scores) were more likely to undergo axillary surgery (95.6% vs. 86.2%, *p* = 0.018; **Supplementary Table S4**), and a similar pattern was observed for IADL (97.5% vs. 90.2%, *p* = 0.023; **Supplementary Table S4**). Patients with better physical status (lower ASA scores) were also more likely to undergo axillary surgery (97.4% vs. 90.4%, *p* = 0.027; **Supplementary Table S4**).

### 3.4 Adjuvant treatment decision by MDT according to age and GA

Overall, 184 patients were recommended at least one adjuvant treatment modality after MDT discussion, and 175 patients commenced at least one adjuvant treatment. MDT recommendations for radiotherapy and chemotherapy differed significantly across age subgroups (**Table 1**). Radiotherapy recommendation rates decreased from 94.1% in the 60–69 year group to 71.4% in patients aged ≥80 years (*p* = 0.017), and chemotherapy recommendation rates fell from 54.0% to 13.6% (*p* < 0.001).

**Table 1:** Multidisciplinary team (MDT) treatment advice on adjuvant therapy according to age group in older patients with breast cancer.

MDT advice on adjuvant therapy	Cases, *n* (%)	Groups			*p*-value
	Total	60–69 years	70–79 years	≥80 years	
Radiotherapy^a^					
Yes	102 (92.7)	48 (94.1)	44 (97.8)	10 (71.4)	**0.017**
No	8 (7.3)	3 (5.9)	1 (2.2)	4 (28.6)	
Chemotherapy^b^					
Yes	89 (42.2)	47 (54)	39 (38.2)	3 (13.6)	**<0.001**
No	122 (57.8)	40 (46)	63 (61.8)	19 (86.4)	
Endocrine therapy^c^					
Yes	148 (87.1)	60 (85.7)	69 (85.2)	19 (100)	0.234
No	22 (12.9)	10 (14.3)	12 (14.8)	0 (0)	
Anti-HER2 therapy^d^					
Yes	15 (78.9)	7 (87.5)	7 (70)	1 (100)	0.675
No	4 (21.1)	1 (12.5)	3 (30)	0 (0)	

Percentages for each modality were calculated among patients for whom that modality was discussed or recommended: radiotherapy, *n* = 110; chemotherapy, *n* = 211; endocrine therapy, *n* = 170; anti-HER2 therapy, *n* = 19.

^a^ Radiotherapy analyzed when *T* ≥ 3 or N+ or having breast conserving surgery (*n* = 110).

^b^ Chemotherapy was discussed for all patients (*n* = 211).

^c^ Endocrine therapy analyzed in ER+ or PR+ patients (*n* = 170).

^d^ Anti-HER2 therapy analyzed in HER2+ patients (*n* = 19).

Significant *p*-values (<0.05) are in bold.

Regarding GA, there was a significant difference in advice on radiotherapy based on scores of VES-13, BI, and ADL. Patients with lower VES-13 scores (better status) were more frequently recommended radiotherapy (95.7% vs. 77.8%, *p* = 0.021). Similarly, higher rates of radiotherapy recommendation were seen among patients with higher BI scores (better status) (96.7% vs. 75%, *p* = 0.003), as well as those with higher ADL scores (better status) (96.3% vs. 83.9%, *p* = 0.035). MDT recommendations for adjuvant chemotherapy also varied according to VES-13, BI, and ECOG-PS status. Patients with lower VES-13 scores (better status) were more likely to receive chemotherapy suggestions (46.8% vs. 21.1%, *p* = 0.004). A similar pattern was observed for higher BI scores (better status) (45.6% vs. 27.5%, *p* = 0.037). Chemotherapy recommendation rates did not follow the same pattern for performance status: patients with ECOG PS ≥2 were more frequently recommended chemotherapy (62.1% vs. 39.0%, *p* = 0.020). All above-mentioned GA-related comparisons are illustrated in **Figure 2**. No statistically significant differences in recommendations for endocrine therapy or anti-HER2 therapy were detected across GA-defined subgroups.

**Figure 2:**
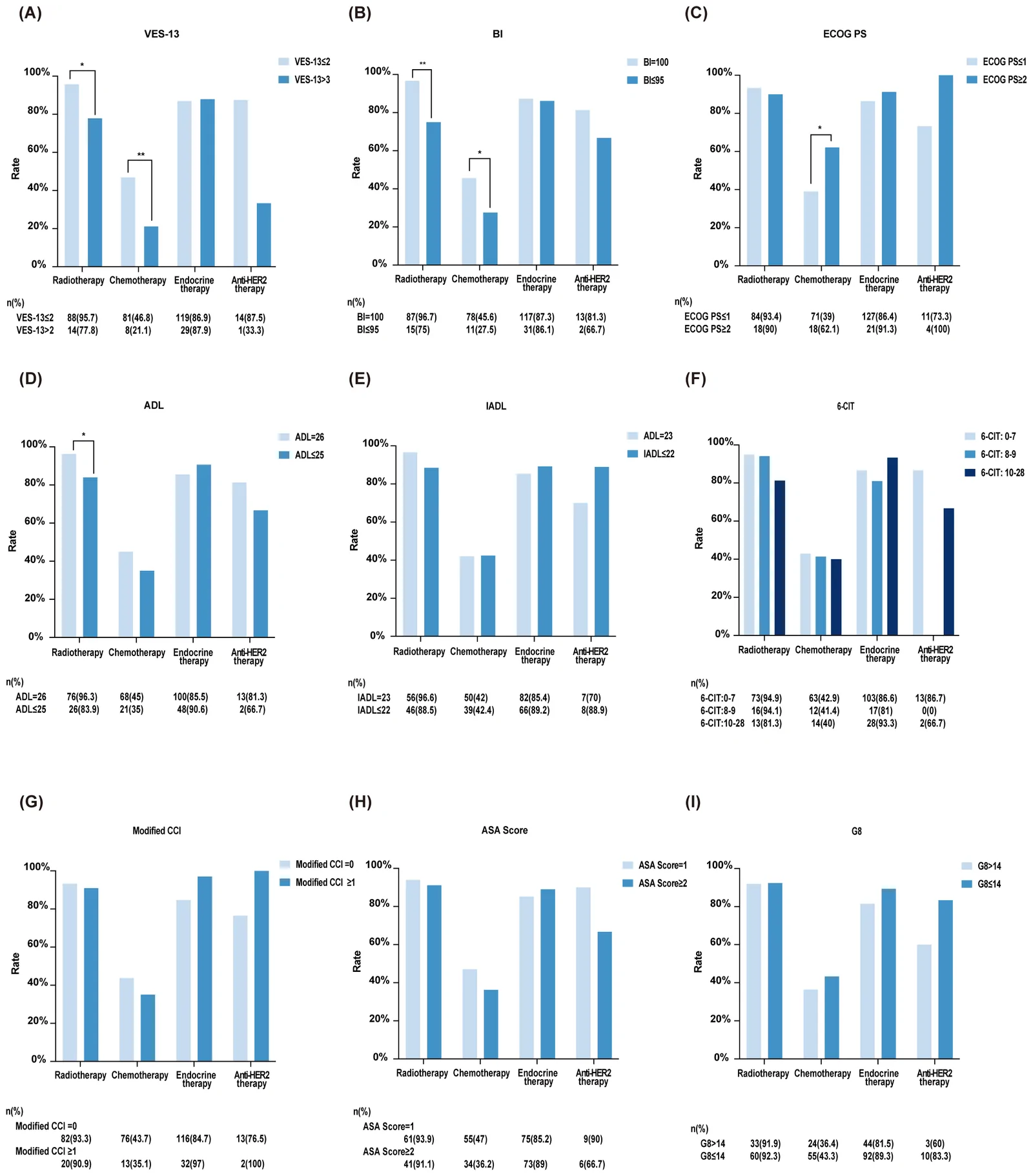
Geriatric assessment (GA) parameters and multidisciplinary team (MDT) treatment recommendations for radiotherapy, chemotherapy, endocrine therapy, and anti-HER2 therapy **(A–I)**. Radiotherapy analyzed in patients with T3 or N+ or having breast conserving surgery. Endocrine therapy analyzed in ER+ or PR+ patients. Anti-HER2 therapy analyzed in HER2+ patients. *p*-values were calculated using Fisher’s exact test. *p ≤ 0.05, **p ≤ 0.01. VES-13, Vulnerable Elders Survey; BI, Barthel Index; ECOG PS, Eastern Cooperative Oncology Group Performance Status; ADL, Activities of Daily Living; IADL, Instrumental Activities of Daily Living; 6-CIT, 6-Item Cognitive Impairment Test; CCI, Charlson’s Comorbidity Index; modified CCI, CCI without age points; ASA Score, American Society of Anesthesiologists Score; G8, Geriatric-8.

### 3.5 Concordance with the MDT recommendation

The concordance with the MDT recommendation in different age groups is displayed in **Table 2**. In general, 82.1% of patients were concordant with the recommended adjuvant therapies. A numerical decline in global concordance was observed across age groups, although this did not reach statistical significance (89.5%, 77.0%, and 76.2% in the 60–69, 70–79, and ≥80 year groups, respectively; *p* = 0.089; **Table 2**). Concordance with radiotherapy decreased significantly with age (100.0%, 81.8%, and 70.0%; *p* = 0.003; **Table 2**), as did concordance with endocrine therapy (98.3%, 89.9%, and 84.2%; *p* = 0.043; **Table 2**). No significant age-group differences were detected for concordance with chemotherapy or anti-HER2 therapy recommendations.

**Table 2:** Concordance with the multidisciplinary team (MDT) recommendation, by age group.

MDT advice concordance	Cases, *n* (%)	Groups			*p*-value
	Total^e^	60–69 years	70–79 years	≥80 years	
Radiotherapy^a^					
Concordance	91 (89.2)	48 (100)	36 (81.8)	7 (70)	**0.003**
Non-concordance	11 (10.8)	0 (0)	8 (18.2)	3 (30)	
Chemotherapy^b^					
Concordance	68 (76.4)	38 (80.9)	28 (71.8)	2 (66.7)	0.569
Non-concordance	21 (23.6)	9 (19.1)	11 (28.2)	1 (33.3)	
Endocrine therapy^c^					
Concordance	137 (92.6)	59 (98.3)	62 (89.9)	16 (84.2)	**0.043**
Non-concordance	11 (7.4)	1 (1.7)	7 (10.1)	3 (15.8)	
Anti-HER2 therapy^d^					
Concordance	14 (93.3)	7 (100)	6 (85.7)	1 (100)	1.000
Non-concordance	1 (6.7)	0 (0)	1 (14.3)	0 (0)	
Overall^e^					
Concordance	151 (82.1)	68 (89.5)	67 (77)	16 (76.2)	0.089
Non-concordance	33 (17.9)	8 (10.5)	20 (23)	5 (23.8)	

^a^ Concordance with radiotherapy was assessed among the 102 patients for whom the MDT recommended radiotherapy.

^b^ Concordance with chemotherapy was assessed among the 89 patients for whom the MDT recommended chemotherapy.

^c^ Concordance with endocrine therapy was assessed among the 148 patients for whom the MDT recommended endocrine therapy.

^d^ Concordance with anti-HER2 therapy was assessed among the 15 patients for whom the MDT recommended anti-HER2 therapy.

^e^ Overall concordance indicates that the actual treatment plan was the same as the one advised by MDT.

Significant *p*-values (<0.05) are in bold.

The relationship between GA scores and treatment concordance was also analyzed. There was no significant difference between overall concordance and GA scores (**Table 3**). Among the individual adjuvant treatments, better concordance with chemotherapy was observed only in patients with lower modified CCI scores (80.3% vs. 53.8%, *p* = 0.038; **Figure 3**). Concordance with radiotherapy, endocrine therapy, or anti-HER2 therapy did not differ significantly according to GA scores.

**Table 3:** Geriatric assessment (GA) parameters and concordance with the multidisciplinary team (MDT)-recommended adjuvant therapy in older patients with breast cancer.

GA parameters	Cases, *n* (%)	Overall^c^		*p*-value
	Total^b^ (*n* = 184)	Concordance (*n* = 151)	Non-concordance (*n* = 33)	
VES-13				
≤2	151 (82.1)	124 (82.1)	27 (81.8)	0.967
≥3	33 (17.9)	27 (17.9)	6 (18.2)	
BI				
100	149 (81)	120 (79.5)	29 (87.9)	0.244
≤95	35 (19)	31 (20.5)	4 (12.1)	
ECOG PS				
≤1	157 (85.3)	128 (84.8)	29 (87.9)	0.647
≥2	27 (14.7)	23 (15.2)	4 (12.1)	
ADL				
26	129 (70.1)	106 (70.2)	23 (69.7)	0.955
≤25	55 (29.9)	45 (29.8)	10 (30.3)	
IADL				
23	101 (54.9)	85 (56.3)	16 (48.5)	0.414
≤22	83 (45.1)	66 (43.7)	17 (51.5)	
6-CIT				
0-7	128 (69.6)	110 (72.8)	18 (54.5)	0.104
8-9	25 (13.6)	19 (12.6)	6 (18.2)	
10-28	31 (16.8)	22 (14.6)	9 (27.3)	
Modified CCI Score				
0	148 (80.4)	124 (82.1)	24 (72.7)	0.218
≥1	36 (19.6)	27 (17.9)	9 (27.3)	
ASA Score				
1	101 (54.9)	80 (53)	21 (63.6)	0.265
≥2	83 (45.1)	71 (47)	12 (36.4)	
G8^a^				
>14	52 (31.1)	44 (31.7)	8 (28.6)	0.748
≤14	115 (68.9)	95 (68.3)	20 (71.4)	

VES-13, Vulnerable Elders Survey; BI, Barthel Index; ECOG PS, Eastern Cooperative Oncology Group Performance Status; ADL, Activities of Daily Living; IADL, Instrumental Activities of Daily Living; 6-CIT, 6-Item Cognitive Impairment Test; CCI, Charlson’s Comorbidity Index; modified CCI, CCI without age points; ASA Score, American Society of Anesthesiologists Score; G8, Geriatric-8.

^a^ G8 unknown in 17 patients engaged in MDT.

^b^ Total analyzed when MDT suggested the necessities of therapies.

^c^ Overall concordance indicates that the actual treatment plan was the same as the one advised by MDT.

**Figure 3:**
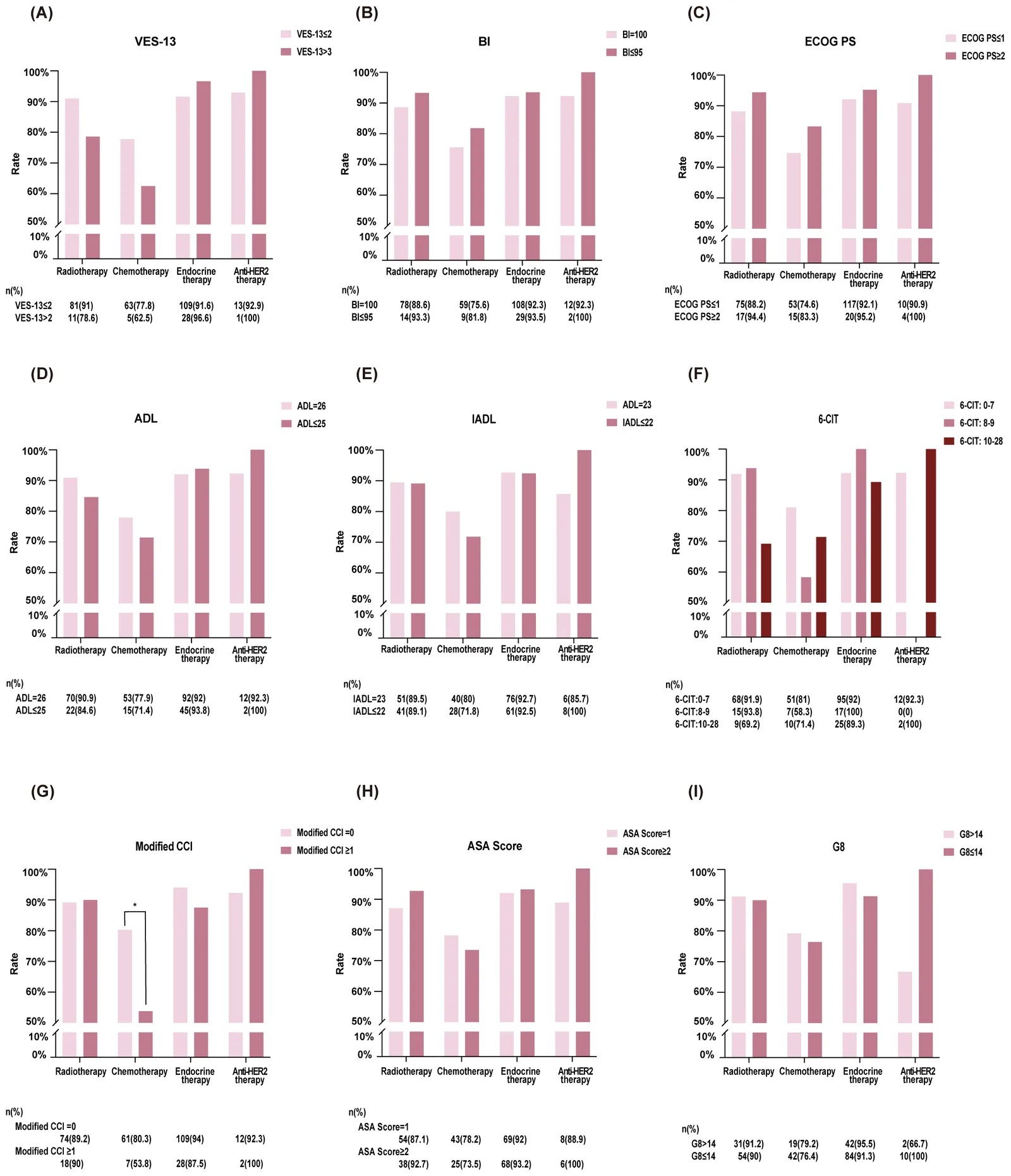
Concordance of the treatment received with the multidisciplinary team (MDT)-recommended adjuvant treatment, according to geriatric assessment (GA) parameters. **(A–I)** were analyzed when MDT suggested the necessities of therapies. *p*-values were calculated using Fisher’s exact test. *p ≤ 0.05. Abbreviations: VES-13, Vulnerable Elders Survey; BI, Barthel Index; ECOG PS, Eastern Cooperative Oncology Group Performance Status; ADL, Activities of Daily Living; IADL, Instrumental Activities of Daily Living; 6-CIT, 6-Item Cognitive Impairment Test; CCI, Charlson’s Comorbidity Index; modified CCI, CCI without age points; ASA Score, American Society of Anesthesiologists Score; G8, Geriatric-8.

## 4 Discussion

This retrospective analysis of a prospectively collected observational cohort included 211 older patients with breast cancer who completed a multidomain GA based on patient-completed questionnaires before curative surgery and attended a postoperative MDT discussion. Patients with lower VES-13 and higher BI scores (both indicating better status) were more likely to be recommended adjuvant radiotherapy or chemotherapy, and patients with lower modified CCI scores (fewer comorbidities) were more likely to be concordant with the recommended adjuvant chemotherapy. Because GA results were not provided to the MDT panel and treatment recommendations were formulated independently of the GA data, these findings represent associations between patients’ health status and treatment recommendations rather than evidence that GA influenced MDT decisions.

In our study, older patients more frequently had higher VES-13 and 6-CIT scores and lower BI, ADL, and IADL scores (all indicating worse status), consistent with previous findings in older patients with breast cancer (32). More than 70% of patients underwent mastectomy, which is higher than in several previous cohorts (33), possibly reflecting patient preference and local surgical practice (34). Axillary surgery was performed in 94.3% (199/211) of patients and ALND was performed in 137/211 (64.9%); 87 patients underwent both SLNB and ALND, 62 underwent SLNB alone, and 50 underwent ALND alone. Patients with lower ADL or IADL scores or higher ASA scores (all indicating worse status) were less likely to undergo axillary surgery, although this did not translate into differences in the adjuvant treatments subsequently recommended; possible explanations include that axillary clearance may not alter subsequent systemic treatment decisions, whereas omitting axillary surgery can preserve arm function and reduce healthcare costs (35).

Adjuvant radiotherapy improves survival through local–regional control in breast cancer, regardless of age (36). Wang et al. reported that 57.6% of patients aged ≥80 years received radiotherapy after breast-conserving surgery and showed that forgoing radiotherapy was associated with higher mortality, even among patients older than 90 years (37). In our cohort, 71.4% of patients aged ≥80 years were recommended adjuvant radiotherapy; this proportion is not directly comparable with the 57.6% reported by Wang et al., because it reflects recommendations rather than treatment receipt and was calculated among patients for whom radiotherapy was discussed. The benefit of adjuvant chemotherapy in older patients remains uncertain (38, 39), and Giordano et al. found that it was limited to patients with lymph node-positive and ER-negative disease (40). In our study, 42.2% of patients overall were recommended adjuvant chemotherapy, and the proportion was considerably lower among patients aged ≥80 years, indicating that further evidence is needed to guide adjuvant chemotherapy in this population. Adjuvant radiotherapy and chemotherapy were more likely to be recommended to patients with lower VES-13 and higher BI and ADL scores (better status), possibly because of the toxicity of these treatments (41). Of note, chemotherapy was recommended more frequently to patients with ECOG PS ≥2 (worse status) than to those with ECOG PS ≤1 (better status). This counterintuitive finding most likely reflects confounding by oncological risk, because treatment recommendations in breast cancer are driven primarily by tumor stage, nodal status, and tumor biology (42, 43), which were not included in these univariable comparisons. In addition, ECOG PS was assigned by the attending clinicians rather than by the questionnaire. This observation is therefore exploratory and should not be interpreted as evidence that poorer performance status favored chemotherapy.

Lack of concordance with MDT recommendations has been associated with a higher risk of recurrence and death (6, 44). In the present study, overall concordance with the recommended adjuvant treatment was 82.1%, which is higher than in some previous reports, possibly because of the MDT support system and the involvement of specialized nurses in follow-up (45, 46). Concordance decreased with advancing age, consistent with the report by van de Water et al. (47). However, this age-related decline was statistically significant only for radiotherapy and endocrine therapy, and not for chemotherapy or anti-HER2 therapy. Among the GA parameters, only a lower modified CCI score (fewer comorbidities) was associated with concordance with the recommended adjuvant chemotherapy. Because the modified CCI specifically captures comorbidity, this finding suggests that comorbidity, rather than functional status alone, may be the main factor influencing whether older patients complete the recommended chemotherapy, possibly through reduced treatment tolerance and competing medical needs (48). As comorbidity frequently coexists with polypharmacy, systematic medication review and timely involvement of the relevant specialists in the MDT may help to make adjuvant regimens safer and more deliverable in this population (49, 50). Because the adjuvant treatments evaluated here are reimbursed under China’s public basic medical insurance scheme, financial barriers to initiation are likely to have been limited, although we did not collect individual financial data. This isolated association should nevertheless be regarded as hypothesis-generating, because the analyses were univariable and were not adjusted for tumor stage, nodal status, or tumor biology, and no correction for multiple comparisons was applied. The composite overall endpoint should also be interpreted cautiously, because patients for whom several modalities were recommended had more opportunities to be classified as non-concordant (51).

Two groups of patients deserve specific comment. Endocrine therapy was not recommended for 22 HR-positive patients; these were predominantly patients with ductal carcinoma *in situ* treated with breast-conserving surgery, in whom endocrine prevention with a SERMestrogen was considered but not recommended because of the risk of venous thromboembolism and cardiovascular events in older patients (52–55). Anti-HER2 therapy was not recommended for four HER2-positive patients, in whom limited disease burden (small tumors) and/or cardiac risk were the main considerations (56, 57). Because these subgroups were very small, they are described descriptively and no statistical inference is drawn from them.

Because our assessment relied on patient-completed questionnaires, cognitive status is directly relevant to its interpretation (58). A considerable proportion of patients had 6-CIT scores in the range suggestive of cognitive impairment. Patients with impaired cognition may not assess their functional status with adequate insight, and their responses should therefore be interpreted with caution (59). In our protocol, specialized breast nurses were present throughout the on-site assessment and assisted participants with item interpretation and answer recording when needed, so that all participants were able to complete the questionnaire-based assessment. Questionnaire completion may be burdensome for some older patients and requires basic digital skills, although on-site nurse guidance likely mitigated digital exclusion. Given the limited number of geriatricians and geriatric nurses, questionnaire-based assessment may reduce the demand on specialized staff (60–62), but evidence on the validity of patient-completed GA in older patients with cancer remains limited, and clinician-rated or caregiver-verified information may still be needed for patients with cognitive impairment (63, 64).

Previous studies in which GA results were actively used by clinicians have shown that such assessment can change cancer treatment plans and reduce chemotherapy-related toxicity (65, 66). In the present study, GA data were collected prospectively within routine clinical documentation but were not provided to the MDT panel and were not part of MDT decision-making; we could therefore examine associations with treatment recommendations rather than the effect of GA on decision-making. In our center, the MDT members were supportive of this study and cooperated throughout the study period, and no stereotypic attitudes toward older patients were observed. Nevertheless, clinicians’ attitudes were not evaluated with a formal instrument, and this remains a relevant barrier to implementation. Future studies should also examine whether artificial-intelligence-assisted administration and interpretation can make GA more feasible and reliable in routine oncology practice.

Several limitations should be acknowledged. First, this was a single-center study with a relatively small sample (211 patients), and participants were recruited consecutively rather than randomly; because all had completed the questionnaire-based assessment, undergone surgery, and attended a postoperative MDT, the cohort is selected and our findings may not apply to frailer patients managed without surgery, in whom GA is often most relevant. Second, GA was performed only once, before surgery, and its results were not provided to the MDT panel; therefore, changes over time and the value of repeated assessment could not be evaluated, and only associations with treatment recommendations, rather than causal effects, could be examined. Third, the analyses were exploratory and predominantly univariable, with multiple comparisons and no formal adjustment for multiple testing; adjusted analyses and effect estimates with confidence intervals were not performed, residual confounding by tumor stage, nodal status, and tumor biology cannot be excluded, and the association between the modified CCI score and chemotherapy concordance should be regarded as hypothesis-generating. Fourth, several potentially relevant variables were not collected (education, social status, family status and living arrangements, medication review and polypharmacy, and the patient-reported burden of assessment); the reasons underlying a discrepancy were not systematically recorded, and initiation, treatment modification, and early discontinuation were not captured as separate endpoints; follow-up was relatively short, so that concordance with endocrine therapy, planned over 5 years, could be assessed only within the available window; in addition, the smartphone mini-program used in this study provides treatment reminders as part of routine care (7), and these platform functions and nurse support may have influenced concordance independently of geriatric status.

In conclusion, in this retrospective analysis of a prospectively collected cohort, age and several multidomain GA parameters (VES-13, BI, ADL, and ECOG PS) were associated with MDT recommendations for adjuvant radiotherapy and chemotherapy in older patients with breast cancer, and a lower modified CCI score was associated with higher concordance with the recommended adjuvant chemotherapy. These findings are exploratory and do not establish that GA influences MDT decisions or treatment outcomes; whether integrating GA into MDT workflows improves decision-making and concordance warrants prospective interventional evaluation.

## Data Availability

The data analyzed in the current study are available from the corresponding authors on reasonable request.
