# Supplementary material for: Association of geriatric assessment with multidisciplinary treatment recommendations and treatment concordance among older patients with breast cancer
Source: Front Oncol. 2026 Oct 20;16:1892469. doi: 10.3389/fonc.2026.1892469 (PMC13727386; doi:10.3389/fonc.2026.1892469)
Supplement: Supplementary file 1 [file DataSheet1.docx]

**Supplementary Materials**

**Supplementary Table S1.** Overview of geriatric assessment scales used in this study

**Supplementary Table S2.** Clinical and pathological characteristics in relation to age

**Supplementary Table S3.** Geriatric Assessment (GA) parameters scores by age group in older breast cancer patients

**Supplementary Table S4.** Geriatric Assessment (GA) parameters scores and association with breast or lymph node surgery in older breast cancer patients

**Supplementary Appendix S1.** Full item lists of the geriatric assessment questionnaires

| **Supplementary Table S1.** Overview of geriatric assessment scales used in this study | | | | | | |
| --- | --- | --- | --- | --- | --- | --- |
| Scale | | Scale score range | Interpretation of higher scores | Cut‑off source for dichotomization |  |  |
| VES‑13 | 0-10 | | Worse status | Published reference(1) |  |  |
| BI | 0-100 | | Better status | Study cohort median |  |  |
| ECOG PS | 0-5 | | Worse status | Published reference(1) |  |  |
| ADL | 0-26 | | Better status | Study cohort median |  |  |
| IADL | 0-23 | | Better status | Study cohort median |  |  |
| 6‑CIT | 0-28 | | Worse status | Original scale recommendation |  |  |
| Modified CCI Score | 0-6 | | Worse status | Study cohort median |  |  |
| ASA score | 0-5 | | Worse status | Study cohort median |  |  |
| G8 | 0-17 | | Better status | Original scale recommendation |  |  |
| Abbreviations: VES-13=Vulnerable Elders Survey; BI=Barthel Index; ECOG PS=Eastern Cooperative Oncology Group Performance Status; ADL=Activities of Daily Living; IADL=Instrumental Activities of Daily Living; 6-CIT=6-Item Cognitive Impairment Test; CCI=Charlson’s Comorbidity Index; modified CCI=CCI without age points; ASA Score=American Society of Anesthesiologists Score; G8=Geriatric-8. | | | | | |  |

| **Supplementary Table S2.** Clinical and pathological characteristics in relation to age | | | | | |  |
| --- | --- | --- | --- | --- | --- | --- |
| Parameters | Cases, n (%) | Groups | | | P value |  |
|  | Total (n=211) | 60-69 yrs (n=87) | 70-79 yrs (n=102) | ≥80 yrs (n=22) |  |  |
| **Pathologic type** |  |  |  |  |  |  |
| IDC | 117(55.5) | 46(52.9) | 57(55.9) | 14(63.6) | 0.672 |  |
| ILC | 10(4.7) | 6(6.9) | 3(2.9) | 1(4.5) |  |  |
| Others | 84(39.8) | 35(40.2) | 42(41.2) | 7(31.8) |  |  |
| **Histologic Grade** |  |  |  |  |  |  |
| I | 14(6.6) | 4(4.6) | 9(8.8) | 1(4.5) | 0.789 |  |
| II | 96(45.5) | 40(46) | 45(44.1) | 11(50) |  |  |
| III | 37(17.5) | 16(18.4) | 19(18.6) | 2(9.1) |  |  |
| N/A^a^ | 64(30.3) | 27(31) | 29(28.4) | 8(36.4) |  |  |
| **Tumor Size** |  |  |  |  |  |  |
| ≤2cm | 126(59.7) | 55(63.2) | 61(59.8) | 10(45.5) | 0.316 |  |
| >2cm | 85(40.3) | 32(36.8) | 41(40.2) | 12(54.5) |  |  |
| **Lymph node involvement** | |  |  |  |  |  |
| Negative | 117(55.5) | 48(55.2) | 58(56.9) | 11(50) | 0.141 |  |
| Positive | 94(44.5) | 39(44.8) | 44(43.1) | 11(50) |  |  |
| **ER expression** |  |  |  |  |  |  |
| Negative | 41(19.4) | 17(19.5) | 21(20.6) | 3(13.6) | 0.740 |  |
| Positive | 170(80.6) | 70(80.5) | 81(79.4) | 19(86.4) |  |  |
| **PR expression** |  |  |  |  |  |  |
| Negative | 56(26.5) | 24(27.6) | 28(27.5) | 4(18.2) | 0.623 |  |
| Positive | 155(73.5) | 63(72.4) | 74(72.5) | 18(81.8) |  |  |
| **HER2 expression** |  |  |  |  |  |  |
| Negative | 192(91) | 79(90.8) | 92(90.2) | 21(95.5) | 0.756 |  |
| Positive | 19(9) | 8(9.2) | 10(9.8) | 1(4.5) |  |  |
| **Ki-67 expression** |  |  |  |  |  |  |
| <14% | 94(44.5) | 39(44.8) | 44(43.1) | 11(50) | 0.840 |  |
| ≥14% | 117(55.5) | 48(55.2) | 58(56.9) | 11(50) |  |  |
| Abbreviations: IDC=invasive ductal carcinoma; ILC=invasive lobular carcinoma; ER=estrogen receptor; PR=progesterone receptor; HER2=human epidermal growth factor receptor 2.  ^a^ N/A for histologic grade corresponds to special breast cancer histological subtypes (including invasive lobular carcinoma, mucinous carcinoma, tubular carcinoma), for which Nottingham histologic grading is not routinely performed in clinical pathology practice. | | | | | |  |
|  |  |  |  |  |  |  |

| **Supplementary Table S3.** Geriatric Assessment (GA) parameters scores by age group in older breast cancer patients | | | | | | |
| --- | --- | --- | --- | --- | --- | --- |
| GA parameters | Cases, n (%) | Groups | | | *P* value | |
|  | Total (n=211) | 60-69 yrs (n=87) | 70-79 yrs (n=102) | ≥80 yrs (n=22) | |  |
| **VES-13** |  |  |  |  |  | |
| ≤2 | 173(82) | 82(94.3) | 83(81.4) | 8(36.4) | **<0.001** | |
| ≥3 | 38(18) | 5(5.7) | 19(18.6) | 14(63.6) |  | |
| **BI** |  |  |  |  |  | |
| 100 | 171(81) | 78(89.7) | 84(82.4) | 9(40.9) | **<0.001** | |
| ≤95 | 40(19) | 9(10.3) | 18(17.6) | 13(59.1) |  | |
| **ECOG PS** |  |  |  |  |  | |
| ≤1 | 182(86.3) | 75(86.2) | 91(89.2) | 16(72.7) | 0.126 | |
| ≥2 | 29(13.7) | 12(13.8) | 11(10.8) | 6(27.3) |  | |
| **ADL** |  |  |  |  |  | |
| 26 | 151(71.6) | 71(81.6) | 73(71.6) | 7(31.8) | **<0.001** | |
| ≤25 | 60(28.4) | 16(18.4) | 29(28.4) | 15(68.2) |  | |
| **IADL** |  |  |  |  |  | |
| 23 | 119(56.4) | 59(67.8) | 53(52) | 7(31.8) | **0.004** | |
| ≤22 | 92(43.6) | 28(32.2) | 49(48) | 15(68.2) |  | |
| **6-CIT** |  |  |  |  |  | |
| 0-7 | 147(69.7) | 68(78.2) | 65(63.7) | 14(63.6) | **0.045** | |
| 8-9 | 29(13.7) | 9(10.3) | 19(18.6) | 1(4.5) |  | |
| 10-28 | 35(16.6) | 10(11.5) | 18(17.6) | 7(31.8) |  | |
| **Modified CCI Score** |  |  |  |  |  | |
| 0 | 174(82.5) | 76(87.4) | 82(80.4) | 16(72.7) | 0.203 | |
| ≥1 | 37(17.5) | 11(12.6) | 20(19.6) | 6(27.3) |  | |
| **ASA Score** |  |  |  |  |  | |
| 1 | 117(55.5) | 52(59.8) | 54(52.9) | 11(50) | 0.554 | |
| ≥2 | 94(44.5) | 35(40.2) | 48(47.1) | 11(50) |  | |
| **G8^a^** |  |  |  |  |  | |
| >14 | 66(34.2) | 30(36.6) | 32(36) | 4(18.2) | 0.211 | |
| ≤14 | 127(65.8) | 52(63.4) | 57(64) | 18(81.8) |  | |
| Abbreviations: VES-13=Vulnerable Elders Survey; BI=Barthel Index; ECOG PS=Eastern Cooperative Oncology Group Performance Status; ADL=Activities of Daily Living; IADL=Instrumental Activities of Daily Living; 6-CIT=6-Item Cognitive Impairment Test; CCI=Charlson Comorbidity Index; modified CCI=CCI without age points; ASA Score=American Society of Anesthesiologists Score; G8=Geriatric-8. Significant P values (<0.05) are in bold. ^a^ G8 unknown in 18 patients. | | | | | | |

| **Supplementary Table S4**. Geriatric Assessment (GA) parameters scores and association with breast or lymph node surgery in older breast cancer patients | | | | | | | |
| --- | --- | --- | --- | --- | --- | --- | --- |
| GA parameters | Cases, n (%) | Breast surgery | | P value | Lymph node surgery | | P value |
|  | Total (n=211) | Mastectomy (n=150) | Breast conserving surgery (n=61) |  | Yes (n=199) | No (n=12) |  |
| **VES-13** |  |  |  |  |  |  |  |
| ≤2 | 173(82) | 121(80.7) | 52(85.2) | 0.433 | 165(82.9) | 8(66.7) | 0.188 |
| ≥3 | 38(18) | 29(19.3) | 9(14.8) |  | 34(17.1) | 4(33.3) |  |
| **BI** |  |  |  |  |  |  |  |
| 100 | 171(81) | 121(80.7) | 50(82) | 0.827 | 163(81.9) | 8(66.7) | 0.222 |
| ≤95 | 40(19) | 29(19.3) | 11(18) |  | 36(18.1) | 4(33.3) |  |
| **ECOG PS** |  |  |  |  |  |  |  |
| ≤1 | 182(86.3) | 133(88.7) | 49(80.3) | 0.111 | 174(87.4) | 8(66.7) | 0.073 |
| ≥2 | 29(13.7) | 17(11.3) | 12(19.7) |  | 25(12.6) | 4(33.3) |  |
| **ADL** |  |  |  |  |  |  |  |
| 26 | 151(71.6) | 108(72) | 43(70.5) | 0.826 | 146(73.4) | 5(41.7) | **0.018** |
| ≤25 | 60(28.4) | 42(28) | 18(29.5) |  | 53(26.6) | 7(58.3) |  |
| **IADL** |  |  |  |  |  |  |  |
| 23 | 119(56.4) | 85(56.7) | 34(55.7) | 0.902 | 116(58.3) | 3(25) | **0.023** |
| ≤22 | 92(43.6) | 65(43.3) | 27(44.3) |  | 83(41.7) | 9(75) |  |
| **6-CIT** |  |  |  |  |  |  |  |
| 0-7 | 147(69.7) | 102(68) | 45(73.8) | 0.559 | 140(70.4) | 7(58.3) | 0.672 |
| 8-9 | 29(13.7) | 23(15.3) | 6(9.8) |  | 27(13.6) | 2(16.7) |  |
| 10-28 | 35(16.6) | 25(16.7) | 10(16.4) |  | 32(16.1) | 3(25) |  |
| **Modified CCI Score** |  |  |  |  |  |  |  |
| 0 | 174(82.5) | 126(84) | 48(78.7) | 0.358 | 164(82.4) | 10(83.3) | 0.935 |
| ≥1 | 37(17.5) | 24(16) | 13(21.3) |  | 35(17.6) | 2(16.7) |  |
| **ASA Score** |  |  |  |  |  |  |  |
| 1 | 117(55.5) | 85(56.7) | 32(52.5) | 0.577 | 114(57.3) | 3(25) | **0.027** |
| ≥2 | 94(44.5) | 65(43.3) | 29(47.5) |  | 85(42.7) | 9(75) |  |
| **G8^a^** |  |  |  |  |  |  |  |
| >14 | 66(34.2) | 41(30.4) | 25(43.1) | 0.087 | 62(34.3) | 4(33.3) | 0.948 |
| ≤14 | 127(65.8) | 94(69.6) | 33(56.9) |  | 119(65.7) | 8(66.7) |  |
| Abbreviations: VES-13=Vulnerable Elders Survey; BI=Barthel Index; ECOG PS=Eastern Cooperative Oncology Group Performance Status; ADL=Activities of Daily Living; IADL=Instrumental Activities of Daily Living; 6-CIT=6-Item Cognitive Impairment Test; CCI=Charlson Comorbidity Index; modified CCI=CCI without age points; ASA Score=American Society of Anesthesiologists Score; G8=Geriatric-8. Significant P values (<0.05) are in bold. ^a^G8 unknown in 18 patients. | | | | | | | |

1.	Okonji DO, Sinha R, Phillips I, Fatz D, Ring A. Comprehensive geriatric assessment in 326 older women with early breast cancer. Br J Cancer. 2017;117(7):925-31.<http://doi.org/10.1038/bjc.2017.257>

**Supplementary Appendix S1**

**Full item lists of the geriatric assessment questionnaires**

**A1. Vulnerable Elders Survey-13 (VES-13)**

(Saliba et al., J Am Geriatr Soc 2001;49:1691–9).

| **1. Age** | 75–84 years | 1 |
| --- | --- | --- |
|  | ≥85 years | 3 |
| **2. Compared with other people your age, how would you rate your health?** | Poor | 1 |
|  | Fair | 1 |
|  | Good | 0 |
|  | Very good | 0 |
|  | Excellent | 0 |
| **3. How much difficulty, on average, do you have with the following physical activities? Six items: (a) stooping, crouching or kneeling; (b) lifting or carrying objects as heavy as 4.5 kg; (c) reaching or extending arms above shoulder level; (d) writing, or handling and grasping small objects; (e) walking 250 metres; (f) doing heavy work around the house.** | A lot of difficulty | 1 per item |
|  | Unable to do | 1 per item |
|  | A little difficulty, some difficulty, or no difficulty | 0 |
| **4. Because of your health or physical condition, do you have difficulty with any of the following activities? Five items: (a) shopping for personal items; (b) managing money; (c) walking across a room; (d) doing light housework; (e) bathing or showering.** | Yes — needs help, or does not do it because of health | 4 (if any item) |
|  | No difficulty | 0 |

Scoring note: a maximum of 2 points can be obtained from item 3; 4 points are assigned if any activity in item 4 is reported as difficult.

**A2. Barthel Index (BI)**

Total score 0–100; higher scores indicate greater functional independence (Mahoney & Barthel, Md State Med J 1965;14:61–5).

| **Feeding** | Independent: food is placed in front of the patient; can feed himself/herself and take any necessary assistive device without assistance. | 10 |
| --- | --- | --- |
|  | Needs some assistance. | 5 |
|  | Totally dependent; unable to feed himself/herself. | 0 |
| **Transfer (bed to chair and back)** | Independent in sitting up and transferring safely, with no safety concerns. | 15 |
|  | Needs minor assistance or supervision, or there are safety concerns. | 10 |
|  | Can sit up independently but needs help to get out of bed. | 5 |
|  | Totally dependent. | 0 |
| **Grooming** | Independent in washing hands and face, brushing teeth, combing hair and shaving. | 5 |
|  | Needs assistance. | 0 |
| **Toilet use** | Independent in getting on and off the toilet; does not soil clothing; can manage a bedpan/commode independently. | 10 |
|  | Needs help with clothing or toilet paper; can place/remove a bedpan but needs help to clean it. | 5 |
|  | Totally dependent. | 0 |
| **Bathing** | Independent. | 5 |
|  | Needs assistance. | 0 |
| **Mobility / Walking** | Walks independently for more than 50 metres; may use a walking aid. | 15 |
|  | Walks more than 50 metres with assistance or support. | 10 |
|  | Unable to walk, but can independently propel a wheelchair for more than 50 metres. | 5 |
|  | Unable to walk or to propel a wheelchair. | 0 |
| **Stairs** | Goes up and down stairs safely; may use a handrail or cane. | 10 |
|  | Needs assistance or supervision. | 5 |
|  | Unable to use stairs. | 0 |
| **Dressing** | Independent in putting on and taking off clothes, shoes and socks. | 10 |
|  | Needs assistance but performs more than half of the task. | 5 |
|  | Totally dependent. | 0 |
| **Bowel control** | Continent; can use suppositories or enemas independently if constipated. | 10 |
|  | Occasional incontinence; needs assistance with suppositories. | 5 |
|  | Dependent; needs assistance with bowel management. | 0 |
| **Bladder control** | Continent; patients with spinal cord injury can manage a urinary bag independently. | 10 |
|  | Occasional incontinence; needs assistance with incontinence pads. | 5 |
|  | Dependent; needs assistance with bladder management. | 0 |

Grading: 100, independent; 75–95, mild dependence; 50–70, moderate dependence; 25–45, severe dependence; 0–20, total dependence.

**A3. Eastern Cooperative Oncology Group Performance Status (ECOG PS)**

| **Grade** | **Description** |
| --- | --- |
| **0** | Fully active; able to carry on all pre-disease performance without restriction. |
| **1** | Restricted in physically strenuous activity but ambulatory and able to carry out work of a light or sedentary nature (e.g., light housework, office work). |
| **2** | Ambulatory and capable of all self-care but unable to carry out any work activities; up and about more than 50% of waking hours. |
| **3** | Capable of only limited self-care; confined to bed or chair more than 50% of waking hours. |
| **4** | Completely disabled; cannot carry on any self-care; totally confined to bed or chair. |
| **5** | Dead. |

Source: Oken MM, Creech RH, Tormey DC, et al. Am J Clin Oncol 1982;5:649–55.

**A4. Activities of Daily Living (ADL) — Physical Self-Maintenance Scale**

Seven items; total score 0–26 (Lawton & Brody, Gerontologist 1969;9:179–86).

| **1. Toilet** | Cares for self at toilet completely; no incontinence. | 4 |
| --- | --- | --- |
|  | Needs to be reminded, or needs help in cleaning self, or has rare (weekly at most) accidents. | 3 |
|  | Soiling or wetting while asleep, more than once a week. | 2 |
|  | Soiling or wetting while awake, more than once a week. | 1 |
|  | No control of bowels or bladder. | 0 |
| **2. Feeding** | Eats without assistance. | 4 |
|  | Eats with minor assistance at meal times, or with help preparing food or cleaning up after meals. | 3 |
|  | Feeds self with moderate assistance and is untidy. | 2 |
|  | Requires extensive assistance for all meals. | 1 |
|  | Does not feed self at all and resists efforts of others to feed him/her. | 0 |
| **3. Dressing** | Dresses and undresses and selects clothes from own wardrobe. | 4 |
|  | Dresses and undresses self with minor assistance. | 3 |
|  | Needs moderate assistance in dressing or selection of clothes. | 2 |
|  | Needs major assistance in dressing but cooperates with efforts of others to help. | 1 |
|  | Completely unable to dress self and resists efforts of others to help. | 0 |
| **4. Grooming (neatness, hair, nails, hands, face, clothing)** | Always neatly dressed and well-groomed, without assistance. | 4 |
|  | Grooms self adequately, with occasional minor assistance (e.g., in shaving). | 3 |
|  | Needs moderate and regular assistance or supervision in grooming. | 2 |
|  | Needs major assistance in grooming but cooperates with efforts of others to help. | 1 |
|  | Actively negates all efforts of others to maintain grooming. | 0 |
| **5. Physical ambulation** | Goes about grounds or city. | 4 |
|  | Ambulates within residence or about one block distant. | 3 |
|  | Ambulates with assistance of another person, a railing, a cane, a walker or a wheelchair. | 2 |
|  | Sits unsupported in a chair or wheelchair but cannot propel self without help. | 1 |
|  | Bedridden more than half the time. | 0 |
| **6. Bathing** | Bathes self (tub, shower, sponge bath) without help. | 4 |
|  | Bathes self, with help in getting in and out of the tub. | 3 |
|  | Washes face and hands only, but cannot bathe the rest of the body. | 2 |
|  | Does not wash self but is cooperative with those who bathe him/her. | 1 |
|  | Does not try to wash self, and resists efforts to keep him/her clean. | 0 |
| **7. Responsibility for own medication** | Is responsible for taking medication in the correct dosages at the correct time. | 2 |
|  | Takes responsibility if medication is prepared in advance in separate dosages. | 1 |
|  | Is not capable of dispensing own medication. | 0 |

Note: this seven-item version yields a maximum score of 26.

**A5. Instrumental Activities of Daily Living (IADL) — Lawton Scale**

Eight items; total score 0–23 (Lawton & Brody, Gerontologist 1969;9:179–86).

| **1. Ability to use the telephone** | Operates telephone on own initiative; looks up and dials numbers. | 3 |
| --- | --- | --- |
|  | Dials a few well-known numbers. | 2 |
|  | Answers telephone but does not dial. | 1 |
|  | Does not use the telephone at all. | 0 |
| **2. Shopping** | Takes care of all shopping needs independently. | 3 |
|  | Shops independently for small purchases. | 2 |
|  | Needs to be accompanied on any shopping trip. | 1 |
|  | Completely unable to shop. | 0 |
| **3. Food preparation** | Plans, prepares and serves adequate meals independently. | 3 |
|  | Prepares adequate meals if supplied with ingredients. | 2 |
|  | Heats and serves prepared meals, or prepares meals but does not maintain an adequate diet. | 1 |
|  | Needs to have meals prepared and served. | 0 |
| **4. Housekeeping** | Maintains house alone or with occasional assistance (e.g., heavy-work domestic help). | 4 |
|  | Performs light daily tasks such as dish-washing and bed-making. | 3 |
|  | Performs light daily tasks but cannot maintain an acceptable level of cleanliness. | 2 |
|  | Needs help with all home maintenance tasks. | 1 |
|  | Does not participate in any housekeeping tasks. | 0 |
| **5. Laundry** | Does personal laundry completely. | 2 |
|  | Launders small items; rinses socks, stockings, etc. | 1 |
|  | All laundry must be done by others. | 0 |
| **6. Mode of transportation** | Travels independently on public transportation or drives own car. | 4 |
|  |  |  |
|  | Arranges own travel via taxi, but does not otherwise use public transportation. | 3 |
|  | Travels on public transportation when assisted or accompanied by another. | 2 |
|  | Travel limited to taxi or automobile, with the assistance of another. | 1 |
|  | Does not travel at all. | 0 |
| **7. Responsibility for own medication** | Is responsible for taking medication in correct dosages at the correct time. | 2 |
|  | Takes responsibility if medication is prepared in advance in separate dosages. | 1 |
|  | Is not capable of dispensing own medication. | 0 |
| **8. Ability to handle finances** | Manages financial matters independently (budgets, writes cheques, pays rent and bills, goes to the bank, collects and keeps track of income). | 2 |
|  | Manages day-to-day purchases but needs help with banking, major purchases, etc. | 1 |
|  | Incapable of handling money. | 0 |

Note: the maximum score for this scale is 23.

**A6. Six-Item Cognitive Impairment Test (6-CIT)**

Total score 0–28; 0–7 normal, 8–9 mild cognitive impairment, 10–28 significant cognitive impairment (Brooke & Bullock, Int J Geriatr Psychiatry 1999;14:936–40).

| **Question** | **Response** | **Score** |
| --- | --- | --- |
| **1. What year is it?** | Correct | 0 |
|  | Incorrect | 4 |
| **2. What month is it?** | Correct | 0 |
|  | Incorrect | 3 |
| **3. Give the patient an address phrase with five components to remember (e.g., John, Smith, 42, High Street, Bedford).** | Not scored at this step |  |
| **4. About what time is it (within 1 hour)?** | Correct | 0 |
|  | Incorrect | 3 |
| **5. Count backwards from 20 to 1.** | Correct | 0 |
|  | 1 error | 2 |
|  | More than 1 error | 4 |
| **6. Say the months of the year in reverse.** | Correct | 0 |
|  | 1 error | 2 |
|  | More than 1 error | 4 |
| **7. Repeat the address phrase (John, Smith, 42, High Street, Bedford).** | Correct | 0 |
|  | 1 error | 2 |
|  | 2 errors | 4 |
|  | 3 errors | 6 |
|  | 4 errors | 8 |
|  | All wrong | 10 |

**A7. Charlson Comorbidity Index (CCI)**

Conditions and weights (Charlson et al., J Chronic Dis 1987;40:373–83). In the present study the age-related points were removed to obtain the modified CCI score used in the analyses.

| **Weight** | **Condition** |
| --- | --- |
| **1** | Myocardial infarction (history, not ECG changes only) |
|  | Congestive heart failure |
|  | Peripheral vascular disease (includes aortic aneurysm ≥6 cm) |
|  | Cerebrovascular disease: CVA with mild or no residua, or TIA |
|  | Dementia |
|  | Chronic pulmonary disease |
|  | Connective tissue disease |
|  | Peptic ulcer disease |
|  | Mild liver disease (without portal hypertension; includes chronic hepatitis) |
|  | Diabetes without end-organ damage (excludes diet-controlled alone) |
| **2** | Hemiplegia |
|  | Moderate or severe renal disease |
|  | Diabetes with end-organ damage (retinopathy, neuropathy, nephropathy or brittle diabetes) |
|  | Tumour without metastases (exclude if >5 years from diagnosis) |
|  | Leukaemia (acute or chronic) |
|  | Lymphoma |
| **3** | Moderate or severe liver disease |
| **6** | Metastatic solid tumour |
|  | AIDS (not just HIV-positive) |

Note: in the original index, one point is added for each decade of age over 40 years. Abbreviations: AIDS, acquired immunodeficiency syndrome; CVA, cerebrovascular accident; ECG, electrocardiogram; HIV, human immunodeficiency virus; TIA, transient ischaemic attack.

**A8. American Society of Anesthesiologists (ASA) Physical Status Classification**

| **Grade** | **Description** |
| --- | --- |
| **ASA 1** | A normal healthy patient. |
| **ASA 2** | A patient with mild systemic disease. |
| **ASA 3** | A patient with severe systemic disease. |
| **ASA 4** | A patient with severe systemic disease that is a constant threat to life. |
| **ASA 5** | A moribund patient who is not expected to survive without the operation. |

Source: Owens WD, Felts JA, Spitznagel EL Jr. Anesthesiology 1978;49:239–43.

**A9. Geriatric-8 (G8)**

Total score 0–17; a score of ≤14 indicates the need for further geriatric assessment (Bellera et al., Ann Oncol 2012;23:2166–72).

| **Has food intake declined over the past 3 months because of loss of appetite, digestive problems, chewing or swallowing difficulties?** | Severe decrease in food intake | 0 |
| --- | --- | --- |
|  | Moderate decrease in food intake | 1 |
|  | No decrease in food intake | 2 |
| **Weight loss during the last 3 months?** | Weight loss >3 kg | 0 |
|  | Does not know | 1 |
|  | Weight loss between 1 and 3 kg | 2 |
|  | No weight loss | 3 |
| **Mobility?** | Bed or chair bound | 0 |
|  | Able to get out of bed/chair but does not go out | 1 |
|  | Goes out | 2 |
| **Neuropsychological problems?** | Severe dementia or depression | 0 |
|  | Mild dementia | 1 |
|  | No psychological problems | 2 |
| **Body mass index (weight in kilograms / height in square metres)?** | BMI <19 | 0 |
|  | BMI 19 to <21 | 1 |
|  | BMI 21 to <23 | 2 |
|  | BMI ≥23 | 3 |
| **Takes more than three prescription drugs per day?** | Yes | 0 |
|  | No | 1 |
| **In comparison with other people of the same age, how does the patient consider his/her health status?** | Not as good | 0 |
|  | Does not know | 0.5 |
|  | As good | 1 |
|  | Better | 2 |
| **Age?** | >85 years | 0 |
|  | 80–85 years | 1 |
|  | <80 years | 2 |
